# Exploiting Joint-Resident Stem Cells by Exogenous SOX9 for Cartilage Regeneration for Therapy of Osteoarthritis

**DOI:** 10.3389/fmed.2021.622609

**Published:** 2021-02-12

**Authors:** Xiaowei Zhang, Shili Wu, Yong Zhu, Cong-Qiu Chu

**Affiliations:** ^1^Division of Arthritis and Rheumatic Diseases, Oregon Health & Science University, Portland, OR, United States; ^2^Section of Rheumatology, VA Portland Health Care System, Portland, OR, United States; ^3^Vivoscript, Inc., Irvine, CA, United States

**Keywords:** osteoarthritis, cartilage, mesenchymal stem cell, SOX9, regeneration

## Abstract

The lack of effective treatment options for osteoarthritis (OA) is mostly due to the very limited regenerative capacity of articular cartilage. Mesenchymal stem cells (MSCs) have been most extensively explored for cell-based therapy to induce cartilage regeneration for OA. However, current *in vitro* expanded MSC-based approaches have significant drawbacks. On the other hand, osteoarthritic joints contain chondrocyte progenitors and MSCs in several niches which have the potential yet fail to differentiate into chondrocytes for cartilage regeneration. One of the underlying mechanisms of the failure is that these chondrocyte progenitors and MSCs in OA joints are deficient in the activity of chondrogenic transcription factor SOX9 (SRY-type high-mobility group box-9). Thereby, replenishing with exogenous SOX9 would reactivate the potential of these stem cells to differentiate into chondrocytes. Cell-permeable, super-positively charged SOX9 (scSOX9) protein is able to promote hyaline-like cartilage regeneration by inducing chondrogenic differentiation of bone marrow derived MSCs *in vivo*. This scSOX9 protein can be administered into osteoarthritic joints by intra-articular injection. This one-step, cell-free supplement of exogenous SOX9 may harness the regenerative potential of the intrinsic MSCs within the joint cavity to stimulate cartilage regeneration in OA.

## Introduction

Osteoarthritis (OA) is the most prevalent type of arthritis affecting about 20–30% of the US adult population (1). The loss of workforce secondary to physical disability and the cost of management of OA impose a substantial socioeconomic impact (2). No drugs have shown to alter the natural course of the disease or slow down the progression of OA. While the etiology of OA remains poorly understood, it is well recognized that OA is a complex and multifaceted disease with a hallmark of articular cartilage degradation (3) that is resulted from chondrocyte degeneration and destruction of cartilage matrix (4). It has been generally believed that cartilage lacks intrinsic capacity of self-regeneration once it is damaged. However, recent studies revealed that joint tissue contains chondrocyte progenitors and mesenchymal stem cells (MSCs) which can differentiate into chondrocytes for cartilage repair under appropriate conditions. Cartilage is a unique tissue comprising matrix proteins and only one cell type, chondrocytes, which are responsible for production of cartilage matrix and maintenance of cartilage integrity. Therefore, restoration of chondrocyte population is critical in cartilage regeneration.

Chondrocytes originate from mesenchymal stromal cells. Naturally, MSCs have been employed for cartilage repair or regeneration for prevention and treatment of OA in animal models and in human clinical trials (5–8). The common approach to MSC-based therapy for OA is that autologous MSCs are expanded *in vitro* then injected intra-articularly into osteoarthritic joints. It has been demonstrated in animal models that cartilage degradation can be reduced by a single intra-articular injection of these *in vitro* expanded MSCs (7). Furthermore, positive effects of autologous MSCs derived from bone marrow or adipose tissue on cartilage regeneration and joint function improvement have been observed in human clinical trials (8, 9).

While MSC-based therapy for OA is promising, several challenges surrounding the quantity and quality of MSCs must be addressed (10). At least three critical conditions for MSCs must be met for a successful MSC-based therapy: sufficient cell number, survival in the joint and capacity of differentiating into chondrocytes. The *in vitro* expansion of MSCs usually takes several weeks and the MSCs in culture may lose “stemness” (9). After a sufficient number of *in vitro* expanded MSCs is achieved, they are transferred into the arthritic joint. MSCs are commonly delivered by intra-articular injection in culture medium without any carrier. It has been reported that fewer than 5% of injected MSCs actually survived in the joint within days after injection (10, 11). Such poor retention may be attributable to several reasons. Carrier-free injection could lead to a leakage of cell suspension during injection and some MSCs might migrate out of the joint cavity. The switch from culture medium to intra-articular environment reduced the viability of the injected MSCs. Most importantly, arthritic joint cavity is not accommodating to the exogenous MSCs (11). To address this problem, many studies used biomaterial carriers, scaffold or hydrogel to embed the MSCs for protection and retention in the joint (10, 12). However, the re-differentiation of dedifferentiated chondrocytes within hydrogels has been a problem (10). Another important challenge is the source of quality MSCs with chondrogenic potential. A majority of the studies employ MSCs from extra-articular sources such as bone marrow and adipose tissue, but these MSCs are known to be poorly chondrogenic in comparison to joint-resident MSCs (13, 14). Recently, intra-articular injection of allogenic MSCs have been explored based on the thought that MSCs were immune-privileged. However, recent studies have demonstrated that MSCs constitutively express major histocompatibility complex (MHC) class I antigens and are capable of expressing MHC class II upon stimulation by inflammatory cytokines (15). This is particularly relevant since that several inflammatory cytokines such as tumor necrosis factor, interleukin (IL)-1β and IL-6 are present in OA joint and create a hostile environment for MSCs, by stimulating their MHC expression, leading to their rejection by the host, and preventing their differentiation into chondrocytes (16). One argument in favor of the use of allogenic MSCs is their immune suppressive property besides their chondrogenesis capability. Indeed, MSCs can express potent inhibitory molecules but these signals may not be sufficient to fend off host alloimmunity. It has been demonstrated in animal models that transfer of bone marrow-derived, MHC-mismatched MSCs were subsequently rejected by the host before they can differentiate into chondrocytes (17). All these factors will limit the usage of this approach with *in vitro* expanded MSCs for the treatment of OA.

## Native MSCs of the Joint

**Figure 1:**
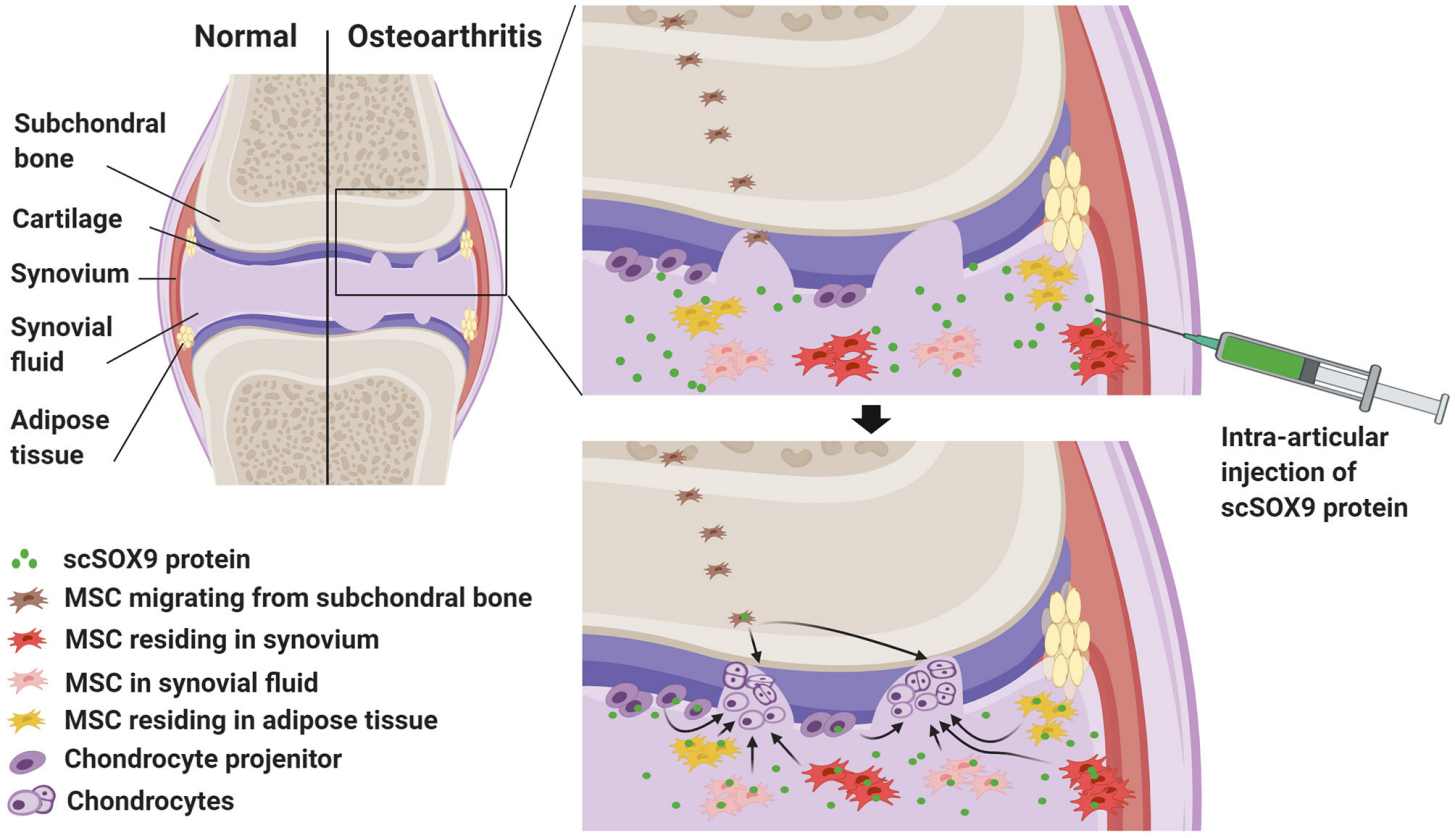
Harnessing joint native mesenchymal stem cells (MSC) by exogenous SOX9 for cartilage regeneration to treat osteoarthritis (OA). This graph highlights cartilage degradation in a joint with OA. In OA joint, there is an increased number of native resident MSCs in synovium, adipose tissue and synovial fluid. These MSCs and chondrocyte precursors are potentially able yet fail to repair the degraded cartilage because of deficiency of chondrogenic transcription factor, SRY-type high-mobility group box-9 (SOX9). Intra-articular injection of cell permeable supercharged SOX9 (scSOX9) fusion protein may drive these stem cells to differentiate into chondrocytes for regeneration of cartilage.

Alternative to implantation of *in vitro* expanded MSCs is motivation and mobilization of native joint-resident MSCs or chondrogenic progenitors for cartilage repair (18). Several anatomical compartments within the joint harbor MSCs which can be potentially directed to differentiate into chondrocytes (Figure 1).

For a considerable long time, articular cartilage is considered a peculiar type of tissue lacking in intrinsic capacity of self-regeneration. Recent studies, on the contrary, revealed evidence that joint tissues including articular cartilage do have mechanisms which can be potentially activated for cartilage regeneration. For instance, Koshino et al. (19) observed biopsy-proven hyaline cartilage regeneration in some patients with knee OA after osteotomy to offload the cartilage defective area of the joint surface. Similarly, by total joint distraction to offload the joint, significant clinical improvement has been achieved in knee OA. The long lasting benefit and the magnetic resonance imaging (MRI) evidence of increased thickness of cartilage suggest articular cartilage regeneration resulted from stimulating the joint endogenous mechanism for cartilage repair (20–23). First, MSC-like resident cell population resides at the cartilage superficial zone (Figure 1) which is important for cartilage tissue homeostasis (24–27). In OA, these MSC-like progenitor cells have been identified (28). It has also long been observed that in the deep zone of cartilage there are MSC-like cells that may be able to migrate and contribute to the chondrocyte clustering in OA cartilage (29, 30) and these cells likely originated from the subchondral bone (Figure 1). Second, MSCs reside in the synovium of the joint. The synovium contains chondrogenic MSCs (Figure 1) accounting for as high as 1% of the cellular components (13, 14, 31). In rabbit models of partial cartilage defect, the MSCs in synovium mobilized to contribute to cartilage repair (32). Third, MSCs reside in joint adipose tissues (Figure 1) (33). Interestingly, MSCs in the synovium and the fat pad can be released spontaneously in a suspended synovium culture model (34). Fourth, synovial fluid contains MSCs (Figure 1). Synovial fluid contains up to 40 MSCs per million mononucleated cells in patients with OA and this number is substantially higher than that in patients with rheumatoid arthritis (35). It is intriguing to note that the number of MSCs in osteoarthritic joint is actually increased in several anatomic regions including the superficial zone of cartilage (28) and subchondral bone (36). In patients with injury of ligaments or meniscus, the number of synovial fluid-resident MSCs increases presumably for initiation of repair (37, 38). Furthermore, endogenous MSCs in synovial fluid are capable of adhering to cartilage in OA distracted joints, probably due to reduced inhibition by synovial fluid hyaluronic acid (39). Importantly, in *in vitro* culture, MSCs isolated from synovial fluid or joint tissue displayed superior chondrogenic potential than those derived from bone marrow or subcutaneous fat (13, 14, 40).

## OA Joint MSCs are Defective in Chondrogenesis and Deficient in SOX9 Function

The question remains to be answered is why these native joint-resident MSCs in OA joint yet fail to repair the damaged cartilage. It is obvious that multiple factors are involved and result in the poor differentiation of these MSCs into chondrocytes. The inflammatory microenvironment or the diseased state of osteoarthritic joints may impair the function of endogenous MSCs, reducing their proliferative and chondrogenic capacity (41). It has been observed that MSC-like progenitor cells in the cartilage superficial zone in OA patients express an early senescent phenotype (28). As a result, the quantity and quality of OA MSC-derived chondrocytes may be less than satisfactory. Previous studies have demonstrated an intrinsic factor which may contribute to the failure of cartilage regeneration in OA, that is, the expression of *S*RY-type high-mobility group box-9 (SOX9) was lower in OA chondrocytes (42–44). Thus, OA chondrocytes expressed a decreased level of SOX9 and the percentage of chondrocytes expressing SOX9 in OA cartilage was significantly lower than that in normal cartilage (42–44). SOX9 belongs to the super gene family of SOX. Many studies have proven that SOX9 is the master transcription factor for chondrogenesis (45). Action of SOX9 is pivotal in chondrogenesis. SOX9 orchestrates transcriptional activation and suppression of many genes involved in cartilage development. In particular, SOX9 co-operates with SOX5 and SOX6 and also upregulates expression of SOX5 and SOX6 genes (46) in induction of chondrogenesis but concomitantly represses RUNX2 to prevent hypertrophy of cartilage (47, 48). Thus, defective SOX9 gene in humans results in campomelic dysplasia (49) with defective development of cartilage. In SOX9 gene knockout mice, embryonic stem cells fail to develop into cartilage (49). Conversely, overexpression of SOX9 gene in MSCs indeed enhanced their chondrogenesis ability (47, 48, 50, 51).

The deficiency of OA chondrocytes may be amended, at least partially, by re-introduction of active SOX9. It has been demonstrated that *in vitro* viral transduction of SOX9 gene in human OA chondrocytes enhances chondrocyte phenotype (52, 53). In isolated and cultured human OA chondrocytes, recombinant adeno-associated virus mediated SOX9 gene overexpression stimulates proteoglycan and collagen type II production (52). Furthermore, in explant cultures of OA articular cartilage, both proteoglycan and collagen type II expression *in situ* was restored to a level comparable to that of the normal cartilage (43).

## Intra-articular Injection of scSOX9 Protein for Therapy of OA

As depicted in Figure 1, since chondrocyte progenitors and MSCs in OA are defective or inadequate in the chondrogenic master transcription factor, SOX9, supplementing exogenous SOX9 will motivate these native joint-resident MSCs or chondrocyte progenitors to differentiate into chondrocytes for cartilage repair or regeneration in OA. Exogenous SOX9 can be supplied by vial transduction (52, 53). However, the vial mediated supplement of SOX9 is not practical for OA treatment. Alternatively, exogenous SOX9 can be supplied as a fusion protein which consists of a super-positively charged green fluorescent protein (GFP) and recombinant human SOX9 with an 11 arginine (11R) tag. This super-positively charged SOX9 (scSOX9) is cell permeable and enters cells to induce MSC differentiation into chondrocytes *in vitro* and *in vivo* (54). Applied at the site of microfracture, scSOX9 was able to promote hyaline-like cartilage regeneration *in situ* by inducing chondrogenesis of bone marrow derived MSCs and the repaired cartilage was durable (54, 55). This scSOX9 protein can be delivered by intra-articular injection into the OA joint. In the joint, scSOX9 will enter MSCs residing in various anatomic compartments of the joint and chondrocyte progenitors at the cartilage surface, and motivate these cells to mobilize to sites of cartilage defects and to differentiate into chondrocytes for cartilage regeneration for therapy of OA (Figure 1).

Theoretically, intra-articularly injected cationic scSOX9 will be naturally attracted to and enriched in the cartilage matrix due to the electrostatic interactions according to the Gibbs-Donnan effect (56). Articular cartilage matrix is highly negatively charged owing to its dense network of collagen fibrils and aggrecan proteoglycans which contain glycosaminoglycan (56). The electrostatic interactions between the cationic scSOX9 and the cartilage matrix will cause a steep concentration gradient at the synovial fluid and cartilage interface. This interaction will also allow retention of scSOX9 within the joint before it exits the synovial fluid. The binding of positively charged scSOX9 and negatively charged articular matrix is weak and reversible, enabling scSOX9 to rapidly penetrate through the full thickness of cartilage. The weak binding is also advantageous for scSOX9 to be released and enter the progenitor cells. It has been shown that a positively charged avidin has a 400-fold higher uptake by cartilage than the neutrally charged avidin (57, 58). In an explant model, positively charged GFP was shown to be accumulated to human and bovine articular cartilage (59). All these findings support the notion that highly positively charged scSOX9 is likely to be concentrated to the cartilage surface.

Several concerns remain with our approach to cartilage repair using scSOX9 for OA treatment and can be investigated in OA models. First, it remains possible but yet to be investigated whether MSCs in the synovium and adipose tissue of the joint will be driven by scSOX9 to differentiate into chondrocytes *in situ*. It is certainly undesirable if chondrogenesis of these MSCs takes place ectopically. However, the chondrogenic potential of synovial and adipose MSCs may not be activated until they are recruited to the cartilage (32). Thus, ectopic chondrogenesis is a concern but may not be present *in vivo*. Secondly, it is not surprising that overexpression of SOX9 in tumor tissue has been observed (60) given the fact that SOX9 is a master transcription factor which is able to maintain cells in undifferentiated status and tends to be elevated during tumor genesis (61, 62). Raising the risk of malignancy by intra-articular injection of excessive exogenous scSOX9 is a legitimate concern, but such concern is lessened by the fact that the articular tissues are exposed to scSOX9 only for a short time in our approach. Lastly, immunity of the host against scSOX9 or the GFP moiety of scSOX9 is another concern since immune response to scSOX9 may lead to its destruction and loss of function and may prevent its re-application in the joint. To reduce the risk of immune rejection, the supercharged GFP moiety can be replaced with a naturally supercharged human protein (63).

In summary, native joint-resident MSCs in several niches are potential cell sources to be differentiated into chondrocytes for cartilage repair or regeneration. These MSCs include those residing in the cartilage surface, synovium, synovial fluid and adipose tissue. Interestingly, the number of these progenitor cells is increased in OA joints (37, 38, 64) but are deficient in the master chondrogenic factor, SOX9. Thus, intra-articular administration of cell-free chondrogenic transcription factor scSOX9 will allow a one-stage and minimal invasive procedure to induce cartilage regeneration by harnessing these intrinsic MSCs within the joint. This represents an innovative and promising approach for treatment of OA.

## Author Contributions

All authors contributed to drafting the manuscript, revising it critically for important intellectual content, and approved the final version to be published.

## Conflict of Interest

SW and YZ are employees of Vivoscript, Inc. which produced scSOX9 used in the previous and ongoing studies. The remaining authors declare that the research was conducted in the absence of any commercial or financial relationships that could be construed as a potential conflict of interest.
